# Arabinose and protocatechuate catabolism genes are important for growth of *Rhizobium leguminosarum* biovar *viciae* in the pea rhizosphere

**DOI:** 10.1007/s11104-015-2389-5

**Published:** 2015-01-30

**Authors:** Paula Garcia-Fraile, Jonathan C. Seaman, Ramakrishnan Karunakaran, Anne Edwards, Philip S. Poole, J. Allan Downie

**Affiliations:** 1 Department of Molecular Microbiology, John Innes Centre, Norwich Research Park, Norwich, NR4 7UH UK; 2 School of Biological Sciences, University of Reading, Reading, RG6 6AJ UK; 3 Present Address: Institute of Microbiology, Academy of Sciences of Czech Republic, Videnska 1083, 142 20 Prague, Czech Republic; 4 Present Address: Department of Plant Sciences, University of Oxford, South Parks Road, Oxford, OX1 3RB UK

**Keywords:** Competitive nodule infection, Legume nodulation, *Pisum*, Rhizosphere fitness

## Abstract

**Background and aims:**

To form nitrogen-fixing nodules on pea roots, *Rhizobium leguminosarum* biovar *viciae* must be competitive in the rhizosphere. Our aim was to identify genes important for rhizosphere fitness.

**Methods:**

Signature-tagged mutants were screened using microarrays to identify mutants reduced for growth in pea rhizospheres. Candidate mutants were assessed relative to controls for growth in minimal medium, growth in pea rhizospheres and for infection of peas in mixed inoculants. Mutated genes were identified by DNA sequencing and confirmed by transduction.

**Results:**

Of 5508 signature-tagged mutants, microarrays implicated 50 as having decreased rhizosphere fitness. Growth tests identified six mutants with rhizosphere-specific phenotypes. The mutation in one of the genes *(araE)* was in an arabinose catabolism operon and blocked growth on arabinose. The mutation in another gene (*pcaM*), encoding a predicted solute binding protein for protocatechuate and hydroxybenzoate uptake, decreased growth on protocatechuate. Both mutants were decreased for nodule infection competitiveness with mixed inoculants, but nodulated peas normally when inoculated alone. Other mutants with similar phenotypes had mutations predicted to affect secondary metabolism.

**Conclusions:**

Catabolism of arabinose and protocatechuate in the pea rhizosphere is important for competitiveness of *R.l. viciae*. Other genes predicted to be involved in secondary metabolism are also important.

## Introduction

Rhizobia are soil bacteria studied primarily because of their ability to infect the roots of leguminous plants, producing nitrogen-fixing nodules. Prior to infecting legume roots, rhizobia grow in the rhizosphere (the region of soil in close proximity to roots) using nutrients secreted from the plant roots. Rhizobia in the rhizosphere are motile and can attach to roots and root hairs where they can grow to form a biofilm. These attached rhizobia are well positioned to detect flavonoids, isoflavonoids and related compounds that induce the bacterial genes required for legume nodulation (Downie 2010).

Each nodule produced on legume roots is usually the result of a clonal infection event and typically a legume such as pea grown in the field produces around 150 nodules (Bourion et al. 2007). This corresponds to about 150 successful infections, but since there can be around 10^4^–10^6^ pea-nodulating bacteria (*Rhizobium leguminosarum* biovar *viciae*) per g of soil (and at least a Kg of soil will be occupied by a mature pea root system), it is clear that the vast majority of *R.l. viciae* bacteria in the soil will not infect peas in any given growing season. Therefore rhizobia must be able to survive and grow well in the rhizosphere, without necessarily infecting legumes.

Although a great deal is known about rhizobial genes required for nodule infection and nitrogen fixation (Downie 2010), less is known about genes required for growth and survival in the rhizosphere, because their identification is relatively difficult. Different approaches have been taken to identify genes required for successful growth in the rhizosphere. For example, promoter trapping approaches (referred to as *in vivo* expression technology, IVET) have been used to identify rhizosphere-expressed promoters in *Pseudomonas fluorescens* (Varivarn et al. 2013) and in *R.l. viciae* (Barr et al. 2008) and a recombination-based variation of IVET has been used to analyse gene expression in *S. meliloti* during rhizosphere and symbiotic growth of *S. meliloti* (Gao and Teplitski 2008). Microarray analyses of bacterial RNA has given insights into genes expressed following growth of *Pseudomonas aeruginosa* in a root exudate (Mark et al. 2005) and of *R.l. viciae* grown in root exudate and in different rhizospheres (Ramachandran et al. 2011). These studies led to the identification of genes induced in these environments and were followed up by the construction of targeted mutations, several of which decreased rhizosphere fitness. An alternative reverse genetics approach has been to use comparative genomics, in an attempt to identify genes prevalent in bacteria that grow in the rhizosphere (Redondo-Nieto et al. 2013; Silby et al. 2009). These reverse genetics approaches can identify many genes likely to be involved in rhizosphere growth or survival, but they require targeted mutagenesis to determine whether the genes are required for the bacteria to be competitive in the rhizosphere.

Direct approaches to identification of mutations that decrease the growth of bacteria in specific environments include signature-tagged mutagenesis (Pobigaylo et al. 2008) and direct sequencing of transposon insertions in large populations of transposon-mutagenized bacteria (Barquist et al. 2013). In both these approaches poorly growing representatives can be identified based on the depletion of bacteria containing specific transposon insertions within a population of bacteria in the tested environment. The depleted bacteria can be detected based on decreased levels of specific transposon insertions identified from parallel DNA sequencing of populations or by using microarrays to identify depletion of tagged transposon insertions in defined groups of mutants. To complement our previous work on IVET selection of rhizosphere-expressed genes (Barr et al. 2008) and microarray analysis and mutagenesis of genes induced in the rhizosphere of peas (Ramachandran et al. 2011), here we have used signature tagged mutagenesis to identify genes which, when mutated, decrease the fitness of *R.l. viciae* in the pea rhizosphere.

## Materials and methods

### Bacterial strains and growth conditions

*R.l. viciae* and *Escherichia coli* strains used in this work are listed in Table 1 *. E. coli* strains were grown at 37 °C in L medium (Sambrook et al. 1989) and *R.l. viciae* strains were grown at 28 °C either in TY medium (Beringer 1974) or in AMS minimal medium containing 10 mM NH_4_Cl and carbon sources as indicated (Poole et al. 1994). Antibiotics were used at the following concentrations (μg ml^−1^): spectinomycin (Spc: 100); streptomycin (Str: 500) and neomycin (Nm: 160) unless specified otherwise. Growth was monitored at 28 °C using an InfiniteF200 microtitre plate shaker/reader (Tecan, Reading, RG7 5AH, UK), measuring absorbance at 600 nm every 40 min. Transductions were done using phage RL38 as described previously (Buchanan-Wollaston 1979). All plant tests were done with *Pisum sativum* cv. Avola peas in a growth chamber at 22 °C with a 16 h-light 8 h-dark light cycle.

**Table 1:** Bacterial strains used in this study

Strain number (mutant ID)	Description features	Source
*R.l. viciae*		
300	WT	(Johnston and Beringer 1975)
Rlv3841	Str^R^ derivative of 300	(Johnston and Beringer 1975)
RU3940	Derivative of 3841 carrying *nif*HΩSpc	Tett *et al.* (2014)
X365 (H1K1F06-1B08)	3841 carrying mTn5 in RL0031	This study
X376 (H2K2B08-1C04)	3841 carrying mTn5 in RL0634	This study
X377(H2K2C06-1B06)	3841 carrying mTn5 in pRL90234	This study
X378H5K5F04-1A10)	3841 carrying mTn5 in RL1109	This study
X379 (H2K2B02-1B05)	3841 carrying mTn5 in RL0811A	This study
X380 (H2K2C07-1C07)	3841 carrying mTn5 in RL4123	This study
X382 (H3K3G11-1E05)	3841 carrying mTn5 in RL3613	This study
X383 (H2K2B09-1B06)	3841 carrying mTn5 in RL0885	This study
X384 (H2K2C07-1E04)	3841 carrying mTn5 in RL3906	This study
X385(H2K2C07-1E03)	3841 carrying mTn5 in RL0079	This study
RU4372	300 carrying mTn5 in RL0031	This study
A1398	300 carrying mTn5 in RL0634	This study
A1399	300 carrying mTn5 in pRL90234	This study
A1401	300 carrying mTn5 in RL0811A	This study
A1402	300 carrying mTn5 in RL4123	This study
A1404	300 carrying mTn5 in RL3613	This study
A1405	300 carrying mTn5 in RL0885	This study
A1406	300 carrying mTn5 in RL3906	This study
*E. coli*		
S17-1 λpir	Donor strain for conjugation of STM plasmids	(DeLorenzo et al. 1993)
XL1-Blue	Supercompetent *E.coli* cells	Stratagene

Bacterial growth in pea rhizospheres was done with pea seedlings grown in 50 ml screw-capped Falcon centrifuge tubes (Fisher Scientific UK Ltd Loughborough LE11 5RG). Washed vermiculite was added to the 30 ml mark on each tube, 10 ml of nitrogen-free FP medium (Fahraeus 1957) was added and the tubes were capped and autoclaved. A single sterile germinating pea seed was added to each tube and after 1 week in a growth chamber, the tubes were inoculated with about 10^4^ bacteria. One week later, bacteria were recovered from the rhizosphere by cutting off the plant shoots, adding 18 ml of phosphate buffered saline, vigorously mixing the tubes for 20 min and then making serial dilutions to count the bacteria. In the absence of pea seedlings there was very little bacterial growth and so the increase in the rhizosphere population (from about 10^4^ to about 10^8^ bacteria) was due to the pea roots.

### Molecular biology techniques

DNA cloning, ligations, transformation, restriction enzyme digestions and DNA hybridizations were done using standard methods (Sambrook et al. 1989). Plasmid DNA was isolated using the Promega (Southampton, SO16 7NS UK) Wizard Plus SV Miniprep DNA Purification System following the recommended protocol. Genomic DNA for DNA hybridization was isolated as described previously (Chen and Kuo 1993). DNA for cloning, PCR, or from the rhizosphere bacteria was isolated using the QIAGEN (UK, Manchester M15 6SH) DNeasy Tissue kit.

Transposon insertion sites were identified either by inverse PCR or arbitrary primed PCR. For inverse PCR, DNA was digested with *Eco*RI and ligated and inverse PCR reactions were carried out using 1 μl ligated genomic DNA, 10 pmol of each of the primers GCGATCCAGACTGAATGCCC and TCGACCTGCATCTAGCCCGC, 7 μl H_2_O, 10 μl Sigma ReadyMix (redtaq PCR mix) Sigma-Aldrich Dorset SP8 4XT, UK) using a PCR protocol as follows; 94 °C for 3 min; 94 °C for 45 s, 58 °C for 45 s, 72 °C for 5 min repeated for 30 cycles. Arbitrary primed PCR was done as described (Das et al. 2005) using the primers GGCCACGCGTCGACTAGTCA-NNNNNNNNNN-CGATC and GGCCACGCGTCGACTAGTCA. Sequencing was done at The Genome Analysis Centre (Norwich UK). Database searches were done at NCBI using the BLAST program (Altschul et al. 1990) and compared to the published sequence of *R.l. viciae* 3841 (Young et al. 2006).

### Construction of signature tagged mutant libraries

A library of pG18-STM plasmids (Pobigaylo et al. 2006) was obtained from Anke Becker from the University of Bielefeld, Germany. These plasmids contain a modified form of mTn*5*-GNm from pCRS487 (Reeve et al. 2006), with additional linker cassettes containing two signature tags and primer binding sites for amplification of the tags. For mutagenesis, each *E. coli* S17-1 λpir carrying a pG18-STM plasmid was cultured at 37 °C in LB Km Gm to OD_600_ 0.4–0.5, mixed (300 μl of *E. coli* and 700 μl of 3841) with freshly TY-grown 3841, the bacteria were pelleted by centrifugation, resuspended in 50 μl TY and spread onto a sterile nitrocellulose filter on a TY plate. Plates were incubated at 30 °C for 40 h, the bacteria on the filters were resuspended in 1 ml AMS and 0.9 ml was inoculated into 10 ml TY Nm, Str and incubated at 26 °C, shaking for 8 h. These bacteria were pelleted by centrifugation and resuspended in 600 μl AMS, 400 μl 50 % glycerol. Prior to storage at −70 °C, 100 μl was plated onto AMS plates containing 10 mM succinate, 20 mM pyruvate, 500 μg/ml Nm, 500 μg/ml Str. After 4 days at 28 °C colonies were picked and stored in 20 % glycerol. Pools of different mutants carrying different tags were arranged as described by Pobigaylo et al. (2006).

### Microarray screening of signature tagged mutants

For each pool of mutants, six pea seedlings were inoculated with a pool of 102 signature-tagged mutant bacteria each with a different tag. Cultures were grown overnight at 26 °C in 10 ml TY Nm Str and 1 ml at OD_600_ 0.1 (approximately 10^8^ CFU) was pelleted, resuspended in 1 ml AMS and diluted (10 μl in 10 ml AMS, to about 10^5^ CFU ml^−1^) and 1 ml of this was inoculated onto to each of six 7-day-old seedlings. Genomic DNA was extracted from the washed bacterial suspension to represent the inoculant pool. A week after inoculation, mutant pools of bacteria from the six rhizospheres were combined into two samples, each derived from the rhizosphere of three plants and DNA was isolated from both samples.

Microarrays were printed onto Corning UltraGAPS slides (Fisher Scientific UK Ltd Loughborough LE11 5RG) by Antony Jones at the Functional Genomics & Proteomics Unit at The University of Birmingham. The signature tags used were 24-mer oligonucleotides. Arrays were printed using a BioRobotics TAS microarraying robot, with eight blocks of 24 × 24 spots. This allows for a minimum of 20 replicas of each experimental tag, and 12 replicas of each “landing light” control tag.

Signature tag oligonucleotides were amplified from genomic DNA isolated from inoculant and rhizosphere bacteria by PCR, using primers fluorescently labelled with 5′ Cy3 or Cy5 marker dyes respectively. PCR reactions for amplifying the signature tags contained 2 μl isolated genomic DNA template, 25 pmol of each primer, 50 μl 2x Promega GoTaq Green Master Mix and made up to 100 μl with H_2_O. The primers STM-P1 (AAAGGACGTGGTTTACGGGGC), STM-P2 (TATATGAATGCCGCCACCCCC), STM-P3 (ATTTTAACTCCCCTCCGCCGC), and STM-P4 TAGTCCTGGTGCATTGAGCCC were as described (Pobigaylo et al. 2006) and the PCR cycle was as described above. The reactions were passed through Macherey-Nagel NucleoSpin Extract II PCR Cleanup kit, and four replica samples were eluted in 74 μl. Samples were eluted into amber Eppendorf 1.5 ml microcentrifuge tubes, to limit the exposure of fluorescently-labelled PCR products to light. The amounts of DNA, and Cy3- or Cy5- incorporation were quantified at 260, 550 and 650 nm respectively.

Aliquots of 5 pmol of each of the Cy3- or Cy5-labelled products were mixed, dried under vacuum, and resuspended in 20 μl H_2_O. Control tags (amplified tags represented on the array but not in the mutant population) were added at a concentration of 500 fmol per control signature tag. Arrays were hybridized using an Advalytix Array Booster DNA Microarray Incubator (Olympus America Inc., Concord, MA, USA).

Microarray slides were scanned using an Axon GenePix 4200A scanner, with GenePixPro 6 software (Molecular Devices Wokingham, RG41 5TS). Slide images were analysed using BlueFuse software, using channel 1 as the control (532, Cy3, inoculant pool) channel and channel 2 as the rhizosphere extract (635, Cy5) channel. BlueFuse output files were imported into the GeneSpring (Agilent Technologies UK, Shropshire SY7 8NR) software package, where replica data for each spot were collated and normalized using Lowess normalizations to show mean values for all oligonucleotide spots. Arrays were compared for Cy3 and Cy5 balance both as a whole and when separated into tag pair groups. Significance was tested using Bonferroni corrected P values as described in the Genespring software.

### Tests of rhizosphere fitness and competitive nodule infection

Fifty mutants showing a statistically significant decrease of at least 2-fold in one tag and at least 1.5 fold decrease in the other tag from the microarrays (Table 2) were mixed with the control strain RU3940 at a ratio (mutant:control) of 10:1 (or 100:1 as specified); strain RU3940 is equally competitive with WT for rhizosphere growth (Tett et al. 2014). To set this up, each strain was grown overnight at 26 °C in a shaking incubator, centrifuged and resuspended in 1 ml of AMS at OD600 0.1. Each mutant (90 or 990 μl) was mixed with the control strain (10 μl) in 10 ml AMS and serially diluted to about 10^4^ CFU per ml and plated in triplicate on selective (Spc or Nm) TY medium to count the bacteria in inoculants. Then 1 ml was inoculated onto each of 9 pea seedlings grown and inoculated as for the mutant screen. After 7 days, rhizosphere bacteria were recovered and the bacteria from three rhizospheres were combined, thereby generating three independent pools each from three pea seedlings. Bacteria in each rhizosphere sample were counted in triplicate using selective (Sp or Nm) TY plates. Only data for those mutants showing significantly poorer growth than the control strain are presented. For those primary mutants that were outcompeted with WT, the mutations were transduced into WT *R.l. viciae* strain 300 and the transductants were retested as described above except that instead of using RU3940 as the competitor strain, 3841 was used. In these experiments the control and mutant bacteria were counted using TY Str or TY Nm respectively; it would have been inappropriate to use RU3940 in the nodule competitiveness tests described below, because it is defective for nitrogen fixation.

**Table 2:** *R.l. viciae* mutants with decreased rhizosphere growth based on microarray analysis of signature tags

Mutant ID (strain number)	Fold change* (Tag 1)	Fold change* (Tag 2)
H1K1 A03-1 D12	2.013	1.639
H1K1 A04-1 B11	2.005	1.826
H1K1 A05-1 B03	2.063	1.576
H1K1 B02-1 A12	2.438	1.557
H1K1 B02-1 C02	2.018	2.380
H1K1 B02-1 C03	2.912	1.552
H1K1 B02-1 C06	2.405	1.836
H1K1 B07-1 C02	2.107	2.704
H1K1 B07-1 C04	2.224	2.685
H1K1 C01-1 C05	4.149	1.779
H1K1 E10-1 D06	2.162	1.986
H1K1 F06-1 B08 (X365)	2.191	2.170
H1K1 G06-1 D01	2.186	1.683
H1K1 H05-1 C02	2.206	1.689
H1K1 H05-1 C04	2.300	1.640
H1K1 H05-1 C10	3.432	1.544
H2K2 B01-1 A12	2.239	1.859
H2K2 B02-1 B05 (X379)	3.584	2.563
H2K2 B09-1 B06 (X383)	2.067	1.928
H2K2 B08-1 C04 (X376)	2.304	1.757
H2K2 B09-1 E05	2.258	1.843
H2K2 C02-1 C06	2.027	3.500
H2K2 C06-1 B06 (X377)	2.707	1.899
H2K2 C06-1 B09	2.105	2.341
H2K2 C07-1 C07 (X380)	2.218	2.736
H2K2 C07-1 E03 (X385)	3.175	2.104
H2K2 C07-1 E04 (X384)	2.011	1.722
H2K2 C12-1 C02	2.104	1.985
H2K2 D07-1 B04	2.764	1.919
H2K2 D08-1 B05	2.003	1.892
H2K2 D10-1 C07	3.026	1.784
H2K2 D11-1 B01	2.353	1.533
H2K2 E03-1 C07	2.324	1.658
H2K2 F08-1 C06	2.162	1.743
H3K3 B08-1 C02	2.092	2.093
H3K3 B08-1 C04	2.101	1.838
H3K3 B08-1 C07	2.240	1.698
H3K3 C07-1 A02	2.290	1.876
H3K3 E11-1 C04	2.597	1.539
H3K3 E11-1 C10	2.153	1.883
H3K3 G08-1 C02	2.399	1.801
H3K3 G11-1 E05 (X382)	2.072	1.509
H5K5 A03-1 C03	2.044	1.875
H5K5 B04-1 D10	2.188	1.795
H5K5 D03-1 C05	2.033	1.610
H5K5 F02-1 A09	2.120	1.940
H5K5 F02-1 A10	2.156	1.631
H5K5 F02-1 D10	2.289	1.968
H5K5 F04-1 A10 (X378)	2.313	1.812
H5K5 H01-1 C03	2.146	1.931

*Fold change is the strength of decrease in Cy5 signal (from rhizosphere bacteria) in putatively rhizosphere impared mutants relative to the Cy3 signal (from the inoculant). All changes shown are significantly different based on a Bonferroni corrected *P*-value = <0.05

Competitive nodule infection experiments were done with inoculants grown as described above except that the control strain was 3841 and mixed with each mutant at a ratio of 1:1. The bacteria (about 10^4^ in 10 ml H_2_O) were inoculated onto germinated pea seedlings in 250 ml flasks containing sand and vermiculite as described previously (Williams et al. 2008). Nodules were collected 25 days after inoculation, surface sterilized and then exudate from crushed nodules was plated on TY Str and TY Nm plates to identify nodule occupancy.

## Results

### Identification of signature-tagged mutants of *R.l. viciae* potentially attenuated for rhizosphere growth

A signature-tagged transposon mutant library was constructed using the pG18-STM plasmids described previously for mini-Tn*5* mutagenesis of *S. meliloti* (Pobigaylo et al. 2008). Each mini-Tn*5* contains two different DNA tags and all of the tags used can be distinguished on the basis of DNA hybridisation. We used a subset of the plasmids to generate pools of mutants and each pool contained 102 mutants all with different mini-Tn*5* insertions. In this approach each mutant is present at a ratio of about 1:100 relative to the population. We used 54 pools of mutants corresponding to 5508 individual mutants and inoculated each pool separately onto the roots of six individual pea seedlings that had been grown for 1 week in sand/vermiculite. A portion of the inoculum was frozen and stored.

Rhizobia were recovered from the rhizosphere a week after inoculation and the signature-tagged oligonucleotides on the mini-Tn*5* insertions were amplified from genomic DNA isolated from both the stored inoculants and from bacteria recovered from the rhizospheres. The DNA was fluorescently labelled using primers labelled with 5′ Cy3 or Cy5 dyes respectively. After clean-up and quantification of DNA and determination of the level of Cy5 or Cy5 incorporation, the labelled DNA was used to hybridise (at high stringency) microarrays containing multiple replicates of the signature tags. Analysis of the data identified 50 mutants that showed a decrease of at least 2-fold in one tag and at least a 1.5-fold decrease in the other tag (Table 2).

### Confirmation of impairment of rhizosphere growth and identification of mutations

**Fig. 1:**
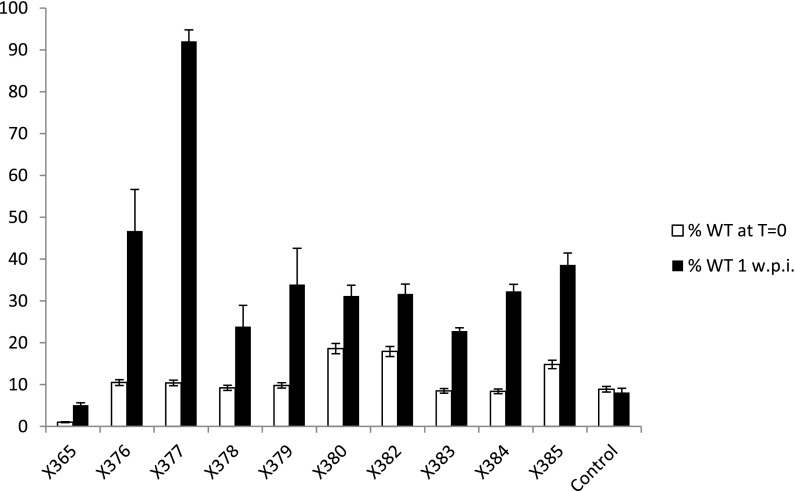
Tests of increased growth of a control strain relative to primary mutants decreased for rhizosphere competitiveness. Mutants identified from microarray experiments as being likely to be defective for rhizoshere competitiveness were mixed with strain RU3940 (which is equally competitive with WT) in a ratio of about 10:1 (except for X365 which was mixed at a ratio of about 100:1) and inoculated into the rhizospheres of one-week-old pea seedlings. After 1 week of growth bacteria were isolated from the rhizospheres. Also shown (control) is a strain which carries mini Tn5 but showed no defect for rhizosphere growth. The *black* and *open bars* respectively show the % of strain RU3940 in the initial inoculum and in the rhizosphere as determined by bacterial counts. The data are averages (± SE) and all comparisons (except the control) were significantly different (*p* = <0.05) based on Tukey’s HSD Test

Each of the 50 mutants identified as being lower in rhizosphere abundance based on the microarray analysis was cultured and mixed in a ratio (10:1 or 100:1) with a Spc-resistant strain (RU3940), which was previously shown to be unaffected for rhizosphere growth in competitive assays (Tett et al. 2014). The ratios of control and tagged mutant bacteria in the inoculants were checked by colony counts selecting with Nm (mutant) or Spc (control) and each mixture of bacteria was inoculated onto multiple pea seedlings grown in sand/vermiculite. After 1 week, the bacteria were recovered from the rhizospheres and dilutions were plated onto TY plates containing Nm or Spc to count the relative numbers of mutant and control bacteria in the rhizospheres. In this assay, the expectation is that if the mini-Tn*5*-tagged mutant is defective for rhizosphere colonisation, then following a week of growth in the rhizosphere, the proportion of the control strain will significantly increase from the level at which it was inoculated. Based on this assay (Fig. 1) ten of the mutants (Table 2) showed a significant increase in the relative numbers of the control bacteria relative to mutants. This confirms the microarray data suggesting that these mutants are less fit for growth in the rhizosphere than the control strain.

The sites of the mini-Tn*5* insertions in the ten mutants were determined by DNA sequencing (Table 3). Nine of the mutations were confirmed to have been caused by insertion of mini-Tn*5*, but one of the mutations (in X385) appeared to have been caused by integration of the whole plasmid carrying mini-Tn*5*. Five of the mutations are in genes associated with intermediate metabolism with the following predicted functions: RL0031, an S-adenosyl homocysteine hydrolase involved in methionine biosynthesis; RL0855, a hydrolase acting on an unknown substrate; RL0634, probably a sugar alcohol dehydrogenase; RL3613, an α-ketoglutarate semialdehyde dehydrogenase; RL4123 a component of a sarcosine oxidase. Two of the mutations are in genes encoding predicted components of transporters, one (pRL90234) an inner membrane permease component and another (RL3906) in a periplasmic solute binding protein, which falls into the family of hydrophobic amino acid uptake transporters (HAAT) and is homologous to *S. meliloti* PcaM involved in protocatechuate transport (MacLean et al. 2008). Two of the mutations appear to be associated with the synthesis of polysaccharides; RL0811A, a predicted lipopolysaccharide glycosyl transferase and RL0079 related to inner-membrane polysaccharide synthases. The mutation in RL1109 is in a gene of unknown function encoding a conserved hypothetical protein.

**Table 3:** Characterisation of mutation sites and doubling times of rhizosphere competitiveness mutants

Strain	Gene mutated	Mutation	Doubling time (h) *	Transductant
3841	WT		5.1 ± 0.1 a	
X365	RL0031 (S-adenosyl-L-homocysteine hydrolase)	mTn5	8.3 ± 0.4 c	RU4372
X376	RL0634 (putative short chain dehydrogenase)	mTn5	4.9 ± 0.1 a	A1398
X377	pRL90234 (Putative permease component of ABC transporter)	mTn5	5.0 ± 0.2 a	A1399
X378	RL1109 (Conserved hypothetical protein)	mTn5	5.1 ± 0.1 a	None
X379	RL0811A (Glycosyl transferase, putative LPS enzyme)	mTn5	5.8 ± 0.1 b	A1401
X380	RL4123 *soxA1* (Putative sarcosine oxidase alpha subunit)	mTn5	5.0 ± 0.1 a	A1402
X382	RL3613 (Arabinose dehydrogenase)	mTn5	5.4 ± 0.1 a,b	A1404
X383	RL0885 (Putative hydrolase)	mTn5	5.1 ± 0.1 a	A1405
X384	RL3906 (Putative solute binding protein for protocatechuate and hydroxy benzoate)	mTn5	4.8 ± 0.1 a	A1406
X385	RL0079 *acsAB* (Putative transmembrane polysaccharide synthase)	*pG18*-STM	8.6 ± 0.3 c	None

*Growth was measured in Y mannitol ammonium medium. Different letters represent significant differences between strains determined using the Tukey-Kramer honestly significant difference test for mean comparison using the SPSS version 19 (IBM Corp. Released 2010. IBM SPSS Statistics for Windows, Version 19.0. Armonk, NY: IBM Corp, USA) Software

### Tests of growth rates of mutants

The selection of the tagged mutants on minimal medium should have excluded auxotrophs, but the mutation in X365 is in RL0031, a gene predicted to be involved in methionine biosynthesis. Therefore, we checked the growth rates of all the mutants in liquid minimal medium (AMS containing 10 mM mannitol). This revealed that X365 (mutation in RL0031) grows more slowly than WT (Table 3); this longer doubling time of 8.3 h was reduced to 6.0 (±0.1) h by the addition of 10 μM methionine. The observation that there was growth in the absence of methionine indicates that the mutation affects, but does not block methionine biosynthesis. The mutation in X385 within a predicted polysaccharide synthase (RL0079) also significantly reduced growth (Table 3). It seems very likely that the reason that these two strains are not competitive in the rhizosphere is due to their poor growth in minimal medium and in the rhizosphere. Most of the other strains grew with growth rates that were not significantly different from WT (Table 3) although X379 carrying a mutation in a predicted LPS glycosyl transferase (RL0811A) also grew slightly slower than WT (Table 3).

### Tests of rhizosphere competitiveness of transductants

The identification of a plasmid integration into RL0079 alerted us to the possibility that although we had identified genes mutated by mini-Tn5 insertions, we could not exclude the possibility of secondary mutations causing the decreased rhizosphere competitiveness. Therefore we transduced the mutations back into the WT (strain 300) and all the transductants were confirmed by PCR to have the mutations in the expected genes. Despite repeated attempts, we were unable to transduce the mutations in the genes RL1109 (strain X378) or RL0079 (strain X385) into WT.

**Fig. 2:**
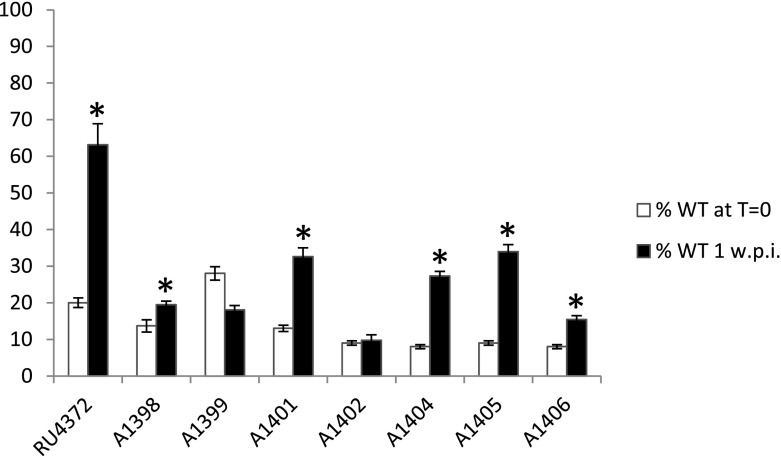
Tests of increased rhizosphere growth of a wild-type strain relative to transductants. Mutations potentially decreasing rhizosphere competitiveness were transduced into *R.l. viciae* strain 300 and the transductants were mixed with the wild-type strain (3841) in a ratio of 10:1 and inoculated into the rhizspheres of one-week-old pea seedlings. After 1 week bacteria were isolated from the rhizospheres. The *black* and *open bars* respectively show the % of the wild-type in the initial inoculum and in the rhizospheres. The data are averages (± SE) and those tests showing significant differences (*p* = <0.05 based on Tukey’s HSD Test) between numbers of the wild-type recovered from the inoculants and rhizospheres are marked with *asterisks*

The eight transductants were tested for growth in pea rhizospheres essentially as described for the original mutants, except that the control strain (present at about 10 % in the inoculum) was 3841. With the exception of A1399, and A1402, the transductants showed a statistically significant decrease in rhizosphere competitiveness based on increased levels of recovery of the control strain (Fig. 2). This demonstrates that mutations in these genes decrease rhizosphere competitiveness. However, there was not significantly enhanced recovery of the control strain relative to the transductants A1399 (carrying the mutation in pRL90234), and A1402 (with the mTn5 inserted in RL4123). Since we confirmed by DNA sequencing that in A1399 and A1402 the mini Tn*5* insertions were in the predicted genes, these mutations appear not to reduce competitiveness in the rhizosphere. This indicates that the rhizosphere defects in the original mutants were probably caused by mutations in other genes.

### Tests of competitiveness for nodule infection

Decreased growth of mutants in the rhizosphere should give rise to decreased competitiveness during nodule infection on the basis that decreased rhizosphere growth should result in reduced numbers associated with the roots and therefore decreased competitiveness for infection. Firstly we tested nodule induction on peas by all the transductants shown in Table 2. None of the mutants tested showed any significant difference in nodulation during the 21 days after inoculation; the plants produced an average of around 70 pink nodules. These observations demonstrate that none of the mutations causing decreased rhizosphere competitiveness blocked nodulation. The presence of pink nodules indicates that the mutants can also reduce nitrogen in nodules and this was confirmed with acetylene reduction assays (data not shown). Since the mutation that we failed to transduce from X385 is in a predicted polysaccharide synthase, and some such mutations can block nodule infection, we also tested this strain for nodulation. X385 formed an average of 60 nodules per plant (not significantly different from WT); again the nodules were pink and of a normal size indicating that the putative polysaccharide made by RL3906 is not required for the symbiosis with pea.

**Fig. 3:**
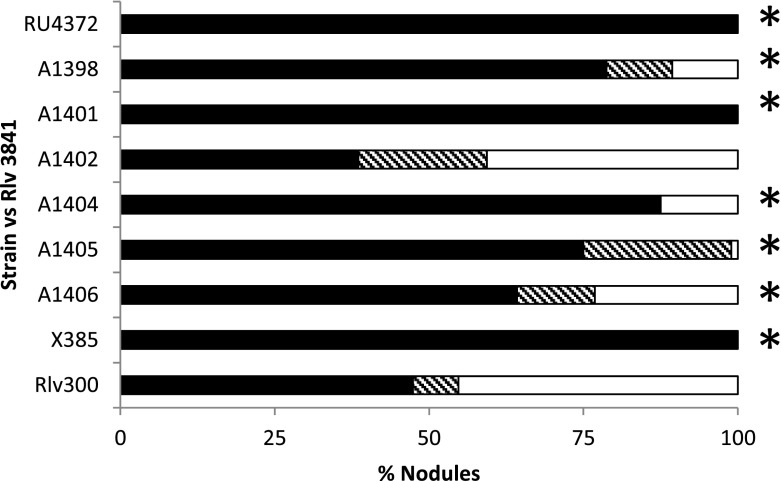
Tests of nodulation competitiveness of mutants with decreased rhizosphere growth. Mutants and the wild-type strain (3841) were mixed 1:1 and inoculated onto pea seedlings grown on sand/vermiculite. Three weeks after inoculation, bacteria were isolated from nodules and the different strains were identified on the basis of their different antibiotic resistances. Mutants showing nodule occupancies significantly lower than expected (chi squared test) are marked with asterisks. Data were obtained using at least three different plants and at least 50 nodules per plant. The *black bars* show the % of nodules occupied by 3841 (Str^R^), the white bars show the % of the nodules occupied by each mutant (Kan^R^) and the *hatched bars* show the % of nodules from which there was growth on both antibiotics

We then tested those transductants (Table 2) that showed a relative decrease in rhizosphere growth for competitiveness in nodule infection tests. Each mutant was inoculated at a ratio of 1:1 together with the control strain 3841 onto peas and then bacteria were extracted from nodules to determine the proportions of nodules infected by mutant or WT. Strains RU4372, A1398, A1401, A1404, A1405, A1406 and X385 were significantly reduced for frequency of nodule infection compared with WT (Fig. 3). As a control we also analysed competitive nodule infection with A1402 and as would be predicted from the lack of a difference in rhizosphere competition, it was also unaffected for competitive nodule infection (Fig. 3).

Analysis of the gene expression patterns using data published previously (Ramachandran et al. 2011) revealed that gene RL0031 (mutated in X365 and RU4372) was not specifically induced in the rhizosphere and so in line with our earlier conclusion, the decreased infection competitiveness of RU4372 is most probably due to the reduced growth caused by partial methionine auxotrophy. Similarly, the putative LPS biosynthesis gene RL0811A (mutated in X39 and A1401) appeared not to be induced in the rhizosphere, and so the decreased rhizosphere and nodule infective fitness of this strain may be caused by the reduced growth rate (Table 3). In contrast, RL0634 (X377 and A1399) was induced in both pea and sugarbeet rhizospheres, RL1109 (X378), RL0079 (X385) and RL4123 (X380 and A1402) were all induced in pea rhizospheres, while RL0085 (X383 and A1404) was induced by pea root exudate (Ramachandran et al. 2011). These patterns of gene expression are in concordance with potential rhizosphere fitness reductions caused by these mutations in genes of undefined functions. The analysis of the genes of defined functions is described below.

### Characterisation of arabinose and protocatechuate utilisation mutants

**Fig. 4:**
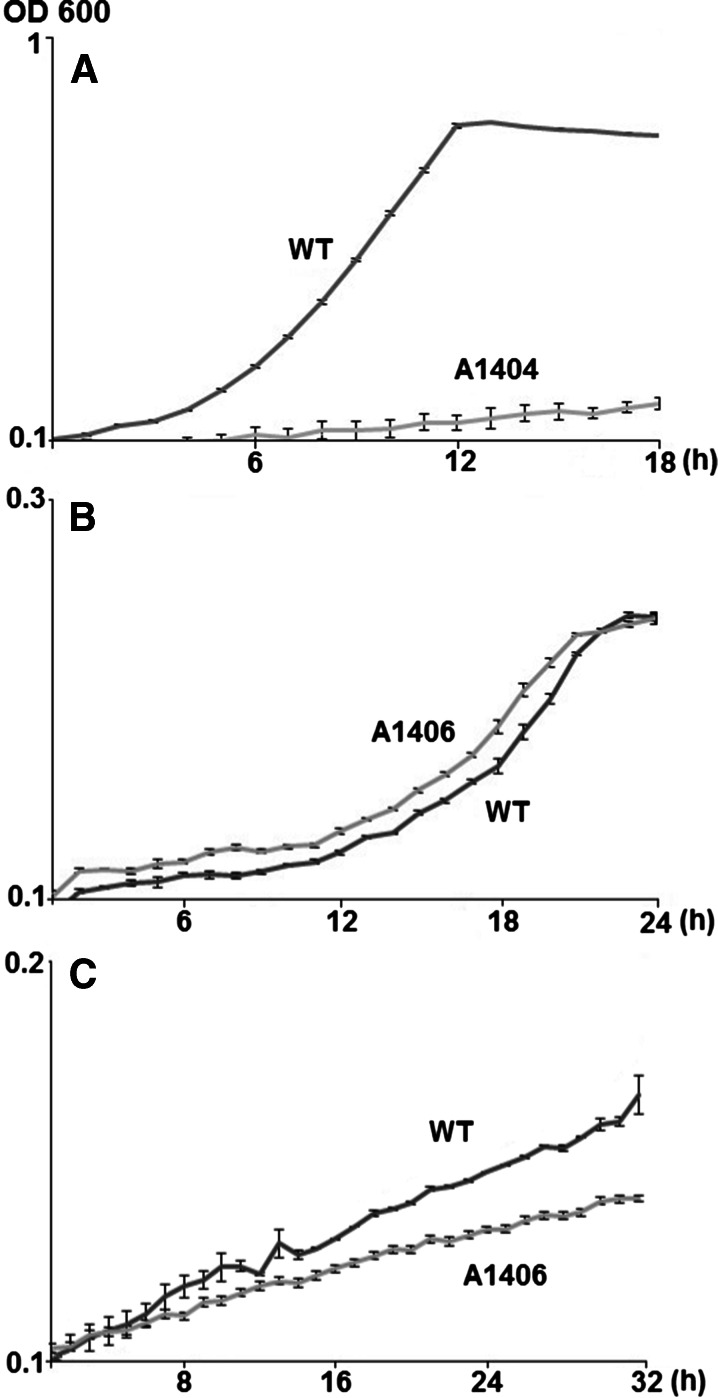
Mutants A1404 or A1406 are defective for growth on arabinose or protocatechuate respectively. **a**: Strains 3841 (WT) and A1404 *(araE)* were inoculated into AMS medium containing 10 mM arabinose. **b**: Strains 3841(WT) and A1406 *(pcaM)* were inoculated into AMS medium containing 5 mM p-hydroxybenzoate **c**: Strains 3841(WT) and A1406 *(pcaM)* were inoculated into AMS medium containing 3 mM protocatechuate. Strains were precultured to stationary phase on AMS mannitol medium, washed and inoculated to an OD600 of about 0.1 into the growth medium containing defined sole sources of carbon. Growth was measured at 28 °C in microtitre plates was monitored at 600 nm every 40 min. The data shown are logarithmic plots of average OD_600_ values (± SE) of three cultures derived from different primary inoculants

The product of RL3613 (mutated in strain X382 and its transductant A1404) is homologous (84 % identity) to AraE (accession CAC49585) within the *araABCDEF* operon required for arabinose catabolism and uptake in *S. meliloti* 1021 (Poysti et al. 2007). A homologous operon is present in *R.l. viciae* equivalent to RL3617-RL3612 and this operon is induced by arabinose (Ramachandran et al. 2011). The *araE* mutant A1404 (carrying mini-Tn5 in RL3613) was unable to grow on arabinose (Fig. 4). Arabinose catabolism can occur via different pathways in bacteria: e.g., in *E. coli* it is catabolised via sugar phosphorylation by being isomerised to ribulose, which is phosphorylated to ribulose−5-phosphate, which is then epimerised into xylulose−5-phosphate. However an alternative pathway occurs in *Azospirilum brasilense* in which arabinose is catabolised sequentially via D-arabinose-1,4 lactone, D arabinoate and 2-dehydro-3-deoxy-D arabinoate to α-ketoglutaric semialdehyde, the last of these steps being catalysed by an enzyme referred to as ketoglutarate semialdehyde dehydrogenase (KGSADH) (Watanabe et al. 2006). There are three types of KGSADHs: types I, II and III are involved in the catabolism of arabinose, glucarate/galactarate and hydroxyproline respectively (Watanabe et al. 2007). RL3613 (*araE*) is similar (33 % identity over the full protein) to the *A. brasilense* Type-I KGDASH (Accession BAE94276) that has been shown (Watanabe et al. 2007) to convert α-ketoglutaric semialdehyde to alpha-ketoglutarate with the concomitant reduction of NADP^+^. The resulting alpha-ketoglutarate from this reaction then enters the TCA cycle. The lack of growth of the *araE* mutants X382 and A1404 is presumably due to the loss of this gene product. However, downstream of RL3613 is RL3612 which shows 78 % identity to arabinonate dehydratase (AraC) from *A. brasilense*, and so it is possible that the mutation could be polar on this gene. The upstream genes RL3617-RL3615; *araABD*) are predicted to encode components of an arabinose transporter as demonstrated previously in *S. meliloti* (Poysti et al. 2007).

The product of gene RL3906 (mutated in X384 and its transductant A1406) is 88 % identical to the periplasmic solute binding protein Smb20568 previously identified in *S. meliloti* strain 1021 (MacLean et al. 2011). The corresponding gene is *pcaM* within the *pcaMNVWX* operon involved in the transport of protocatechuate. This operon is regulated by the LysR-type regulator PcaQ whose DNA-binding site is known (MacLean et al. 2008). A PcaQ-like binding site was predicted upstream of RL3906 (MacLean et al. 2011) and the products of the genes downstream of RL3906 (RL3907-RL3910) are all homologous (>75 % identity) to PcaN, PcaV, PcaW and PcaX respectively. Upstream of (and in the same orientation as) RL3906 is RL3905 which is predicted to encode parahydroxybenzoate hydroxylase, that catabolises 4-hydroxybenzoate to protocatechuate. By analogy with *S. meliloti*, all of these genes are probably regulated by the LysR-type transcriptional regulator PcaQ, which is homologous to RL3904. Protocatechuate induced the expression of RL3906 and the genes RL3095-RL3910 were all induced by 4-hydroxybenzoate (Ramachandran et al. 2011). Furthermore, the gene most strongly induced in WT *R.l. viciae* (strain 3841) in the pea rhizosphere is RL3016 encoding a predicted protocatechuate oxidoreductase (Ramachandran et al. 2011), indicating that this pathway is important for rhizosphere fitness. Growth of *R.l. viciae* WT on protocatechuate as sole C source was poor in WT and was significantly reduced in the mutant (A1406) carrying the mutation in *pcaM* (RL3906) (Fig. 4). We tested growth on p-hydroxybenzoic acid as sole carbon source; unlike *S. meliloti* strain 1021 we observed growth on this substrate, but we saw no difference between the *pcaM* (RL3906) mutant A1406 and WT.

## Discussion

There are some important issues that came to light in this study aimed at identifying rhizobial genes important for rhizosphere growth. One is that it may be important to determine that the identified mutation caused the observed phenotype; one of the cleanest phenotypes was seen with the primary mutants X377 and X380, but the phenotype was not seen with the transductants A1399 and A1402, indicating that the identified mutations did not cause the rhizosphere phenotype. Using phage-mediated transduction of the mutations into the wild-type has two advantages: (a) it demonstatrates that the transposon caused the phenotype of decreased rhizosphere competitiveness and (b) it reconfirms the phenotype in completely independent tests. Relatively few of the previous signature-tagged-mutagenesis screens have confirmed that the genes identified were caused by the transposon insertions that were characterised. This issue of secondary mutations potentially leading to false conclusions is not a concern in the saturation mutagenic approaches used to analyse mutants decreased for growth in specific environments.

Another issue was that there can be significant variability in the bacterial counts and sometimes these may obscure real differences caused by the mutations. In this regard, we believe that measurements of the nodule infection gave good corroboration, because decreased growth in the rhizosphere would be predicted to reduce the rhizosphere population of mutants, thereby decreasing their probability of infecting nodules. Clearly assays of competitive nodule infection reflect more than rhizosphere colonisation, but it would be surprising if a mutant decreased for rhizosphere growth nodulated at equivalent levels to WT. Therefore, although initially there appeared to be some decrease of rhizosphere colonisation caused by mutation of RL4123 (a predicted sarcosine oxidase), the observation that A1402 carrying the mutation was unaffected for competitive nodule infection suggests that this gene is not critical for rhizosphere growth. This contrasts with e.g., strains A1398 and A1406 where the rhizosphere growth decrease is relatively small, but their competitive nodule infection is clearly compromised. The mutations affecting arabinose and protocatechuate utilisation also caused decreased competitive nodule infection.

Arabinose is a likely substrate that could be used by rhizobia in the rhizosphere; it is a component of the plant cell wall and is present in arabinogalactan proteins secreted by plant root cells. Utilising such arabinose would require additional enzymes that would release it from the polysaccharides in which it is found. When a reporter-GFP construct induced by arabinose, was used to determine if arabinose was present on barley roots, induction of the reporter occurred mainly at the root-seed boundary and primary roots, but not at root tips (Casavant et al. 2002). It seems likely that arabinose released directly or indirectly from pea roots can enable *R.l. viciae* 3841 to be more competitive in the rhizosphere, leading to a modest increase in nodule competitiveness. In *S. meliloti*, arabinose transport and catabolism has been analysed and arabinose catabolism mutants nodulated normally on alfalfa; however no significant effect was observed on competitiveness for infection of alfalfa nodules (Poysti et al. 2007).

Whereas arabinose is a good carbon source for rhizobial growth, protocatechuate and 4-hyroxybenzoate are poorer substrates. Protocatechuate is catabolised via the β-ketoadipate pathway in *S. meliloti* (MacLean et al. 2006) and similar genes are present in two clusters in *R.l. viciae* 3841: pRL110086 (*pcaG*); pRL110087 (*pcaH1*) pRL110088 (*pcaC*), pRL110089 (pcaD), pRL110090 (*pcaQ*); and pRL110286 (*pcaR*), pRL110287 (*pcaI*), pRL110288 (*pcaJ*) and pRL110289 *(pcaF*). Although in *S. meliloti* the protocatechuate transporter genes *pcaMNVWX* (Smb20568–Smb20784) are linked to the degradation genes (MacLean et al. 2011), these genes appear not to be linked in *R.l. viciae* (Mauchline et al. 2006). The mutation in RL3609 affects a predicted protocatechuate solute binding protein gene on the chromosome whereas the catabolism genes are on plasmid pRL11. It has been suggested, based on inhibitor studies that there is one transporter that is specific for dihydroxy-benzoic acids (like protocatechuate) and one for mono-hydroxy benzoic acids, like 4-OH benzoate (Wong et al. 1991) and our observation that the mutation in RL3609 affects growth on protocatechuate but not 4-OH-benzoate is consistent with that conclusion. Protocatechuate at concentrations above 5 mM can inhibit growth and so two roles for protocatechuate degradation can be envisaged; one for catabolism of low amounts enabling growth of *R.l. viciae* on protocatechuate and another for degradation of toxic metabolites such as proptocatechuate (or related compounds). Our data indicate that protocatechuate or related metabolites may be important for rhizosphere fitness. One possible source of this substrate is from lignin degradation which can produce metabolites like vanillate and syringate which are further catabolized through the protocatechuate catabolism pathway (Kamimura et al. 2010).

## Conclusions

Screening for mutants of *R.l. viciae* with reduced growth or survival in the pea rhizosphere showed that the ability to catabolise arabinose and protocatechuate is important for rhizosphere fitness. The reduced rhizosphere fitness of these mutants was correlated with decreased nodule infection competitiveness. Other genes of undefined function were also identified as reducing rhizosphere fitness and again this was correlated with decreased competitiveness for nodule infection. .
